# Cancer suppression and the evolution of multiple retrogene copies of TP53 in elephants: A re‐evaluation

**DOI:** 10.1111/eva.13383

**Published:** 2022-04-25

**Authors:** Leonard Nunney

**Affiliations:** ^1^ Department of Evolution, Ecology, and Organismal Biology University of California Riverside Riverside California USA

**Keywords:** elephant, hyrax, manatee, multistage carcinogenesis, Peto’s paradox, retrogene, TP53

## Abstract

Evolving to become bigger and/or longer lived should increase cancer susceptibility, but this predicted increase is not observed, a contradiction named Peto's paradox. A solution is that cancer suppression evolves to minimize cancer susceptibility, and the discovery of 19 retrogene (RTG) copies of the tumor suppressor gene TP53 in the African elephant (*Loxodonta africana*) is increasingly cited as a classic example of such adaptive suppression. However, classic examples need rigorous evaluation and an alternative hypothesis is that the RTGs spread by genetic drift. This study shows that before its duplication, the ancestral elephant RTG was already truncated from 390 amino acids to 157 by a frameshift mutation, and that 14 of the 19 copies are now truncated to ≤88 amino acids. There was no compelling evidence of either positive or negative selection acting on these 88 codons, and the pattern of RTG accumulation fits a neutral model with a duplication rate of ~10^−6^ per generation. It is concluded that there is no evidence supporting the hypothesis that the 19 elephant RTGs spread to fixation by selection; instead, the evidence indicates that these RTGs accumulated primarily by segmental duplication and drift. It is shown that the evolutionary multistage model of carcinogenesis (EMMC) predicts the recruitment of 1–2 independently acting tumor suppressor genes to suppress the increased cancer risk in elephants, so it is possible that one or a few RTGs may have been favored by selection resulting in the known enhanced sensitivity of elephant cells to DNA damage. However, the analysis does not provide any support for either a direct (via conserved TP53 activity) or indirect (via supporting canonical TP53 function) role of the RTGs sequences, so that the presence of multiple copies of TP53 retrogenes in elephants needs to be further justified before being used as a classic example of tumor suppression in large‐bodied animals.

## 1 INTRODUCTION

The multistage model of carcinogenesis (Nordling, 1953) forms the basis of our understanding of how cancers originate. Under this model, cancer initiates once a single cell has accumulated a set of tissue‐specific driver mutations. The probability of this set of mutations accumulating increases with the number of cells in the tissue and the number of cell divisions, hence one of the predictions of this model is that large, long‐lived animals such as humans should have a much higher incidence of cancer than small, short‐lived ones such as mice (Peto, 1977). This increase is not seen (Abegglen et al., 2015; Vincze et al., 2022), and to resolve “Peto's paradox” (the disconnect between observation and the theoretical expectation) Nunney (1999, 2003) introduced the evolutionary multistage model (EMMC) in which cancer suppression is treated as an evolving trait. Under the EMMC, adaptive evolution results in increased cancer suppression in species that have evolved to become larger and/or longer lived. Evidence supporting this view is of two types. First, under the basic multistage model, cancer risk within the freely interbreeding members of a species should increase with body size (or, more specifically, with the number of cells). This prediction is confirmed by the observation that within both humans and domestic dogs, larger individuals (or breeds in the case of dogs) are more prone to cancer (Nunney, 2013, 2018). Second, there is support for the EMMC’s defining assumption that cancer suppression evolves as the body size and/or reproductive life of species changes. Compelling evidence for such adaptive evolution was provided by the discovery that telomerase suppression in fibroblasts of 15 rodent species increased with body size (Seluanov et al., 2007), and that the small but long‐lived naked mole‐rat and the (unrelated) blind mole‐rat both provided evidence of additional (but different) mechanisms of cancer suppression (Gorbunova et al., 2012).

More subtle support for the EMMC was established using worldwide data on the incidence of 10 adult human cancers. The EMMC predicts that selection will act to reduce the fitness loss due to different cancers down to relatively similar levels. This quantitative prediction is hard to test; however, a qualitative prediction is that tissues with high stem cell division rates are expected to have an increasingly elevated incidence of cancer relative to other tissues as age increases, but only during the postreproductive period (when selection is weak). This was precisely the pattern found (Nunney & Thai, 2020).

Both elephants and humans have evolved over time from smaller, short‐lived ancestors, so the EMMC predicts that both taxa have evolved additional anticancer mechanisms. There is a broad range of potential mechanisms (Caulin & Maley, 2011), and the EMMC can be used to predict the degree of change needed to maintain a constant cancer risk (Caulin et al., 2015; Nunney, 1999, 2020). Modeling indicates that the magnitude of changes required in somatic mutation or immune efficiency to completely compensate for large increases in body size and longevity (e.g., from a mouse to a human or a whale) are implausibly large, whereas direct suppression requires only modest increases in the number of tumor suppressor genes involved (Nunney, 2020). One particularly plausible way to achieve this increase is via the duplication of tumor suppressor genes (Nunney, 2003). Given the increasing availability of genomic data, the possible involvement of gene duplication can be tested by searching for duplicated tumor suppressor and other cancer‐related genes in large and/or long‐lived animals (Caulin et al., 2015) such as elephants (Vazquez & Lynch, 2021) and whales (Tejada‐Martinez et al., 2021; Tollis et al., 2019).

A search of the genome of the African elephant (*Loxodonta africana*) resulted in the discovery of 19 retrogene (RTG) copies of TP53, with multiple copies also present in the genome of the Asian elephant (*Elephas maximus*; Abegglen et al., 2015). RTGs are processed duplicate copies of a “parent” (i.e., canonical) gene and they typically lack introns and the cis‐regulatory regions of the parent gene. The RTG copies of TP53 are of particular interest because TP53 encodes the p53 protein, a protein critical in responding to a range of cellular stresses including inducing apoptosis in response to DNA damage, and is one of the most important genes acting to suppress cancer (Levine, 2020). Elephants are expected to have evolved enhanced cancer suppression as they evolved into the large long‐lived mammals that we see today. It, therefore, seemed probable that the multiple RTGs of TP53 were spread and were maintained as a result of selection for enhanced cancer suppression. This conclusion was reinforced by the finding that cells of both elephant species are markedly more sensitive to DNA damage than human cells (Abegglen et al., 2015; Sulak et al., 2016), supporting the hypothesis that the RTGs act by increasing the removal of mutated and hence potentially precancerous cells through apoptosis.

The duplication and spread of RTG copies of TP53 in the elephant genome has rapidly become one of the classic examples used to illustrate the evolution of cancer suppression (Aktipis, 2020), but as such it requires careful validation. Despite the compelling circumstantial evidence favoring this view, there are some concerns, most notably the presence of stop codons in all RTGs, including one that would terminate the protein before the DNA‐binding motif (Sulak et al., 2016). An alternative hypothesis, considered here using a detailed analysis of the genomic data, is that most, and perhaps all, of the RTG duplicates spread by genetic drift and not via selection for cancer suppression.

The origin of the elephant clade was about 60 million years ago (Gheerbrant, 2009), when it separated from the manatee and hyrax clades. Since that time elephants have increased substantially in size and longevity from an ancestor that was probably about the size of the rock hyrax (Gheerbrant, 2009). Prior to the split of the manatee, hyrax, and elephant clades, a single ancestral TP53 RTG had already been inserted into the ancestral genome (Sulak et al., 2016); however, the expansion beyond that single copy occurred only in the elephants, an expansion that seems to have continued steadily over time by the repeated segmental duplication of more than 20 kb of DNA (Sulak et al., 2016). All of the RTGs contain stop codons, but five show some evidence of transcription (Abegglen et al., 2015; Sulak et al., 2016) and at least one (RTG#12) is transcribed at a significant level and may be translated (Sulak et al., 2016). There are a variety of ways in which these truncated mRNAs or proteins could promote the activity of the canonical p53, such as acting as a decoy to allow the canonical TP53 protein to escape negative regulation or degradation (Abegglen et al., 2015; Sulak et al., 2016).

The discussion of the TP53 expansion in the elephant has focused on the hypothesis that the progressive evolutionary increase in body size in the elephant clade resulted in a progressive accumulation of the 19 RTGs, thereby increasing p53 activity, which in turn led to a progressive increase in cancer suppression. The alternative possibility noted above is that duplicate RTGs accumulated via genetic drift. This alternative leaves open the possibility that one or a few RTG copies were subsequently favored by a fortuitous effect of enhancing p53 function.

To shed more light on this question, a number of issues were examined. First, how much added protection against cancer is the elephant expected to evolve? Second, did any of the RTGs ever code for a fully functional p53? Third, is there evidence that the increase in the number of segmental duplicates containing the RTG was favored by selection acting to increase the number of TP53 RTGs? Fourth, is there any evidence that selection has acted within the coding sequence of the RTGs?

## 2 METHODS

The adaptive response predicted by the EMMC, in terms of either the number of tumor suppressor genes recruited or the reduction in the somatic mutation rate, was estimated for the African elephant (*L*. *africana*) and the Florida manatee (*Trichechus manatus latirostris*) assuming that their common ancestor was similar in size and longevity to the rock hyrax (*Procavia capensis*). For comparison purposes, the equivalent estimate was made for humans given a primate ancestor of similar size and longevity to the hyrax. In each case the changes in organ size (and specifically the stem cell population of those organs) were assumed to be proportional to the changes in overall body mass. The response was estimated for three cancers (colorectal, esophageal, hepatocellular) following a previous approach (Nunney, 2020), where the risk for each cancer (*p*) was estimated using the formula derived for the multistage model (Nunney, 1999), which, given small *p*, is defined by:

(1) $$p=C{\left(ukT\right)}^{M}$$

where *C* and *T* reflect, respectively, size and longevity (i.e., proxies for the number of at‐risk stem cells and the total time for cell division). In addition, *u*, *k*, and *M* define the somatic mutation rate, the number of cell division per unit time, and the number of driver mutations required to initiate the cancer. Previous work (Nunney, 2020) used the house mouse as the reference point, and here the approach was identical except that the hyrax was the reference and reproductive lifespan instead of total lifespan was used. Reproductive lifespan (from birth to the cessation of reproductive effort, including parental care) rather than the total lifespan was used because it better reflects the period over which selection for cancer suppression can act.

For each cancer, using Equation (1), an integer value of *M* was combined with an appropriate somatic mutation rate (*u*) to give the hyrax a (reproductive) lifetime cancer risk (*p*) of 0.1%. This risk is roughly consistent with the incidence of most human cancers at age 55 years. Using these baseline parameters, it was determined how much change in either *u* or *M* was needed in the other species to maintain a 0.1% cancer risk. For each species, the values of *C* and *k* used were derived from human estimates (Nunney, 2020), either directly (*k*) or scaled by the weight of each species (*C*), using average weights obtained from panTHERIA (Jones et al., 2009). Estimates of reproductive lifespan (*T*) were obtained for hyrax (Hoeck, 1989), manatee (Marmontel et al., 1997), and for human and elephant (Lahdenperä et al., 2014).

It has been proposed that the well‐established decrease in metabolic rate per unit weight (Kleiber's law; Kleiber, 1947) may ameliorate the expected increase in cancer risk with body size (Dang, 2015). Although it appears that this effect can, at most, play a relatively minor role, a possible effect on the cell division rate with a maximum scaling of C^−0.15^ could not be dismissed based on the available data (Nunney, 2020). Therefore, this possibility was also considered, with the division rate decreasing with increased weight relative to the reference (hyrax) according to (C/C_hyrax_)^−0.15^.

The DNA sequence data were downloaded from NCBI (https://www.ncbi.nlm.nih.gov/genome) using the relevant genomes (African savanna elephant [GCA_000001905.1], rock hyrax [GCA_004026925.2 and GCA_000152225.2], Florida manatee [GCA_000243295.1], and Asiatic elephant (*Elephas maximus*) [GCA_014332765.1]). The RTG sequences were aligned by eye in BioEdit (Hall, 1999). The resulting alignment is shown in File S1. The scaffolds containing the RTGs were also downloaded for the African elephant, hyrax, and manatee. Using the location of the RTG, each scaffold was trimmed so that the reverse‐complement RTG was at the 5′ start of each sequence. Each sequence was then trimmed at its 3′ end to a maximum of 50 kb (several were much shorter). The sequences were then aligned using Clustal W (Thompson et al., 1994; see File S2). This was done in several rounds of removing and re‐adding sequences because the excessive number of unknown bases (i.e., NNNs) sometimes caused software failure.

The evolutionary relationships among the RTGs were estimated in MEGA X (Kumar et al., 2018) using the GTR + G maximum likelihood model, with the strength of the nodes being tested using 500 bootstrap replications. Using this tree, the evidence for positive selection was examined using codeml in PAML (Yang, 2007). Two types of model were used. First, the branch model, comparing model 0 (one value of dn/ds) to model 1 (a dn/ds for each branch within the tree), was used to determine if there was heterogeneity in dn/ds across the RTG tree. The presence of heterogeneity would trigger an unbiased search for the source of the heterogeneity following the protocol introduced by Nunney and Schuenzel (2006). Second, the sites model, comparing neutral and selection options using NSsites = 1 and 2, was used to detect specific codons showing evidence of being subject to positive selection across the RTG tree. The less stringent models M8 and M8a were also employed for completeness (using a 1 *df* test; Wong et al., 2004).

## 3 RESULTS

### 3.1 The predicted adaptive response to the risk of cancer

The EMMC was used to estimate the magnitude of the adaptive change expected in the evolution from a hyrax‐like ancestor to modern‐day elephant or to a manatee (Table 1). This change was estimated either in terms of tumor suppressor recruitment (i.e., as in increase in *M*) or as a reduction in the somatic mutation rate (*u*). The nature of the evolutionary response was different for different cancers, because some cancers originate in tissues with a large stem cell population that divide either at a relatively high rate (e.g., colorectal cancer) or at a low rate (hepatocellular cancer), while others originate in much smaller stem cell population (esophageal cancer). The need for added tumor suppressor genes was highest in the case of colorectal cancer, requiring *M* ≥ 3.6 which is roughly the equivalent of two additional tumor suppressor genes (since it takes two mutations to disable both copies of a tumor suppressor gene). However, for adaptation to occur exclusively via a reduction in the somatic mutation rate, then a very large reduction (of more than 200‐fold) would be required to control the incidence of hepatocellular cancer. This latter effect arises because the influence of any change in *u* is amplified by the exponent *M* (equation 1), and *M* is lowest for hepatocellular cancer. Incorporating the maximal plausible metabolic effect into the calculation has the effect of reducing the magnitude of the required adaptation but it does not eliminate it (Table 1).

**TABLE 1:** The predicted adaptive increase in cancer suppression needed to control three different cancers in the African elephant and Florida manatee via either an additive increase in driver mutations (*M*) or a multiplicative decrease in somatic mutation rate (*u*) assuming their common ancestor had a body size and life span similar to the rock hyrax

		Parameters		Colorectal cancer				Hepatocellular cancer				Esophageal cancer			
		C (human)		2 × 10^8^				3.01 × 10^8^				6,652,800			
		k (human)		73 divs/year				0.9125 divs/year				33.2 divs/year			
		Baseline *u*		4.0 × 10^−6^		2.5 × 10^−6^		8.2 × 10^−7^		5.2 × 10^−7^		4 × 10^−6^		2.6 × 10^−6^	
		Baseline *M*		4		4		2		2		3		3	
				No MR		MR effect		No MR		MR effect		No MR		MR effect	
		Av weight (kg)	Max age (years)	*M*add	*u*: fold reduce	*M*add	*u*: fold reduce	*M*add	*u*: fold reduce	*M*add	*u*: fold reduce	*M*add	*u*: fold reduce	*M*add	*u*: fold reduce
Rock hyrax	*Procavia capensis*	2.95	11	–	–	–	–	–	–	–	–	–	–	–	–
African elephant	*Loxodonta africana*	3940	65	+3.6	x36	+1.5	x12	+1.1	x217	+0.7	x74	+2.2	x65	+1.3	x22
Florida manatee	*Trichechus manatus*	467	40	+2.3	x13	+1.4	x6	+0.7	x46	+0.5	x21	+1.7	x19	+0.8	x9
Human	*Homo sapiens*	58.7	55	+2.3	x11	+0.9	x7	+0.6	x22	+0.4	x14	+1.6	x14	+0.9	x9

Note

The results assume either no metabolic rate effect of size on division rate (no MR) or an effect scaled by C^−0.15^ (MR effect). The equivalent adaptive change required in humans was estimated assuming an ancestor with a similar size and longevity to the hyrax. Parameters: C = estimated number of stem cells; k = estimated number of divisions. Max age defines the estimated age by which reproduction ceases.

It is, therefore, probable that the elephant has adapted to its larger size and increased reproductive longevity by adding tumor suppressor gene activity. This adaptation could involve increased p53 activity. The manatee is also expected to have adapted, but to a lesser degree than the elephant, and to a degree similar to humans, assuming that the human ancestor had a similar life history to the hyrax (Table 1). In each case, these adaptive changes would have occurred independently in each lineage and hence are likely to be different.

### 3.2 The nature of the ancestral elephant TP53 RTG

Sulak et al. (2016) demonstrated that all of the elephant TP53 RTGs originated from a single RTG present in the common ancestor of the elephants, hyraxes, and manatees. This raises the important question of whether or not this ancestral form still coded for a complete functional form of p53 at the base of the elephant clade. This question was addressed by examining the distribution of deletions in the RTGs, since deletions are uncommon relative to base changes. Specifically, using the 1173‐bp African elephant TP53 sequence as a reference, there are 445 sites where the sequence base differs from the reference in one or more of the 19 RTGs and only 21 deletions, that is, a more than 20‐fold difference. Moreover, these deletions can be accurately aligned with the reference (File S1). There are also 7 insertion events, all of which are small except for one 25‐bp and one 5‐bp insertion.

**FIGURE 1:**
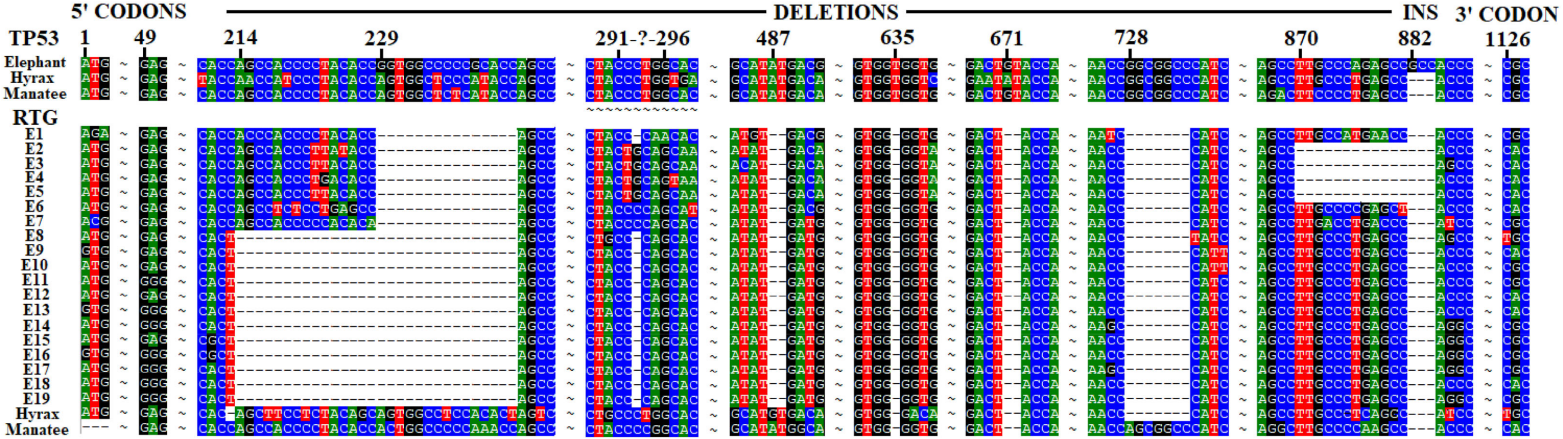
Aligned sequences of TP53 and RTGs of the African elephant, the Florida manatee, and the rock hyrax showing deletions in the RTGs, a single insertion in the elephant TP53, and the codons identified as selected in the 5′ region (1 and 49) and in the pseudogene 3′ region (1126). The gene position is in “ref bp,” that is, from the start of the elephant TP53 sequence

Comparing the single RTG of the rock hyrax (*Procavia capensis*) and the Florida manatee (*Trichechus manatus*) and the 19 RTGs of the African elephant (*L*. *africana*) with their TP53 sequence (and again using the African elephant TP53 as a reference), there is a 1‐bp (bp#635) and a 2‐bp (bp#671–672) deletion shared by all RTG sequences but none of the canonical TP53 sequences (Figure 1). Thus, these two deletions occurred in the single ancestral copy prior to the split of the three clades; however, their effect was potentially minor, since although the local frame shift introduced a novel 12aa sequence between the two deletions, the net 3‐bp deletion meant that the rest of the protein was unaffected.

**FIGURE 2:**
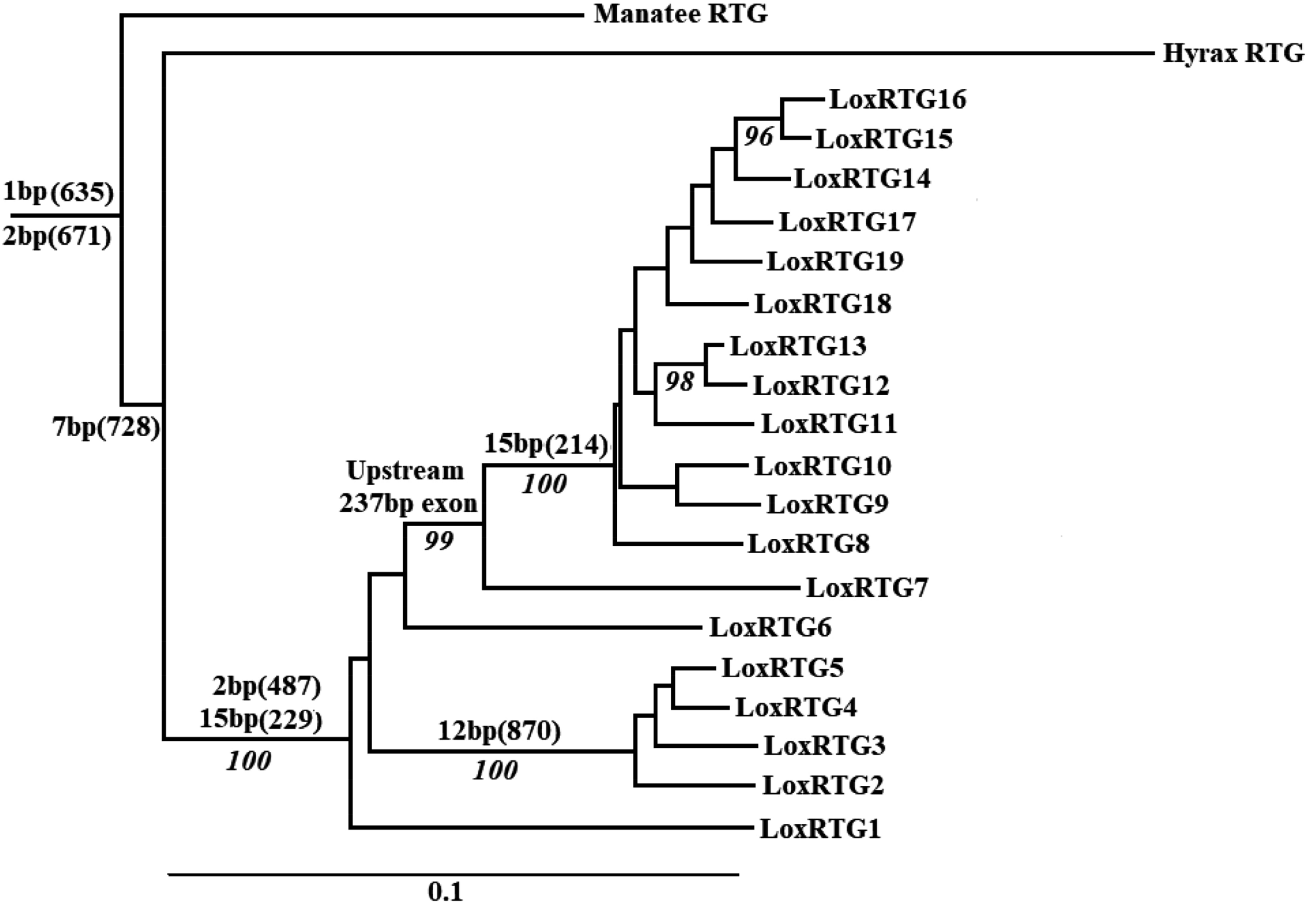
The maximum likelihood tree of the 19 RTGs of the African elephant and the single RTGs of the rock hyrax and Florida manatee, rooted by the canonical TP53 sequences (not shown). Inferred occurrences of indels are shown together with their length and location (in parenthesis; see Figure 1). Bootstrap values greater than 90% are also shown

Another deletion that predated the elephant clade was a 7‐bp deletion shared by the hyrax RTG and all 19 elephant RTGs (bp#728–734; Figure 1). This created a frame shift that resulted in a pair of stop codons (bp#1016–1018 and bp#1022–1024). As a result, the RTG protein sequence was randomly changed starting from amino acid position 242 (aa#243 in the elephant p53) until its premature termination by a stop codon at a length of 334 amino acids. The elephant p53 is 390 amino acids in length, hence the sequence data indicate that the initial single RTG in the elephant clade coded for only 59% of the canonical p53 protein, that is, (241–12)/390, given that 12 amino acids within the “intact” 5′ coding region and all amino acids after position 241 were randomly changed by frame shifts. These basal deletion events are mapped onto the RTG phylogeny (Figure 2).

A 3‐bp insertion in the elephant TP53 (bp#882‐884) appears like a deletion in all RTGs; however, its status as an insertion is confirmed by its absence in the canonical hyrax and manatee sequences (Figure 1).

Following the split of the elephants from the manatee and hyrax lineages, it was estimated to be about 10 million years before a second RTG copy became established (Sulak et al., 2016). Two deletions are uniquely shared by the 19 African elephant RTGs, a 15‐bp deletion (bp#229–243) that removed 5 amino acids from the 5′ (potentially functional) end of the RTG product, and a 2‐bp deletion (bp#487–488) that created a new frame shift (Figure 1) basal to all elephant RTGs (Figure 2). As a result, the p53 coded by the ancestral RTG sequence was randomized from amino acid position 158 until the protein was terminated by a new stop codon at position 167 (ref. bp#519–521). Thus, before any RTG duplication occurred the sequence data indicate that the single ancestral elephant RTG was coding for a protein that was only 40% related to the original p53 protein (i.e., 157 of 390 amino acids).

These deletion patterns were independently verified by examining the three RTGs found in the genome of the Asian elephant, present on scaffolds 731, 1426, and 1637 (File S1). All three contain the 5 deletions basal to the elephant clade (starting at bps #229, #487, #635, #671, #728; see Figure 1).

### 3.3 Evolutionary pattern of the African elephant TP53 RTGs

Figure 2 shows the inferred relationship among the RTGs of the African elephant and the single RTG of the closest relatives of the elephants, the hyrax, and manatee. The relationships are essentially identical to those presented in Sulak et al. (2016) (with their numbering of the 19 elephant RTGs retained), but with branch lengths shown. The relationship of the RTGs, as defined by sequence data, is further supported by their deletions, the most informative of which are shown in Figures 1 and 2. The remaining deletions that involve 3 or more RTGs add to this support: a unique 1‐bp deletion (ref bp#574) unites RTGs #8–19; a unique 1‐bp deletion (ref bp#597) unites RTGS #2–5; and a unique 1‐bp deletion (ref bp#813) uniting RTGS #3–5 (File S1).

After the duplication of the ancestral RTG, further duplications followed, but it is notable that these duplicates were all derived from only one of the initial pair, with RTG#1 never resulting in any additional copies (Figure 2). This may suggest that RTG#1 was the single ancestral copy, with the second copy perhaps moving into a region of the genome that facilitated the subsequent segmental duplications. However, bootstrap support for these initial duplications is <90%, and furthermore detailed analysis of the duplicated sequence around the RTGs was precluded by the limited size, the incomplete nature of the current scaffolds, and the potential movement of transposable elements in and out of the region. However, given those caveats, based on the first 30 kb of upstream sequence the RTG #1 scaffold does have the longest region of high sequence similarity with the equivalent hyrax and manatee region; however, expanding the reference to 50 kb, RTGs #2 and #5 show an additional region of match to the manatee, a region that is absent from the hyrax (Table S1). Given that RTG #4 also shows this same match to the manatee (and the RTG #3 scaffold does not extend that far), the possibility that the initial duplication was an RTG within the clade of #2–5 cannot be excluded. The overall alignment is shown in File S2.

As shown above, the ancestral copy of the elephant RTGs was disrupted by a frame shift starting after amino acid #158 (ref bp#487) which raises the possibility that selection could have favored greater matching to the canonical p53 amino acid sequence. Such selection would lead to the spread of variants that increased the length of the in‐frame product. However, there is no support for this hypothesis since there has been a universal decrease in the in‐frame length. Only RTG#7 has retained close to this ancestral length, becoming scrambled by a frame shift after its codon #157; however, this RTG lacks a methionine start codon (Table S1).

The RTGs #8–19 were all affected by a single base pair deletion somewhere in the range ref bp#291 to 294, with a phylogenetically inconsistent but potentially identical single base pair deletion in RTG #1 in the range ref bp#291 to 296; however, this deletion is unusual in being ambiguous in its location (Figure 1) and hence in its history. The deletion created a frame shift starting with amino acid #88 in RTGs #8–19 (Table S1). This group includes RTG#12 which appears to have some effect in increasing p53 activity (Sulak et al., 2016), which raises the possibility that during the RTG expansion selection may have been acting within the first 88 codon positions.

PAML analysis (Yang, 2007) was used to search for evidence of positive selection within these first 88 codons of the RTGs. The branch model indicated that optimizing the dn/ds ratios for each branch did not provide a better fit than a single dn/ds value (χ^2^ = 39.12 with 34 *df*, *p *= 0.251). Furthermore, this single value (dn/ds = 1.20) was not significantly different from 1.0 (χ^2^ = 0.92 with 1 *df*, *p *= 0.338). These results are consistent with the neutral evolution of a pseudogene. However, the test for positive selection at individual codon sites (PAML model M1 and M2) was highly significant (χ^2^ = 10.66 with 2 *df*, *p *= 0.005), identifying two codons subject to selection (dn/ds > 1), codon #1 (dn/ds = 3.71; *p *= 0.015) and codon #16 (dn/ds = 3.65; *p *= 0.034; see Figure 1). The more permissive test (PAML model M8a and M8) identified the same two codons, but, as expected, with a more apparent confidence (codon #1: dn/ds = 3.13, *p *= 0.007; and codon #16: dn/ds = 3.11, *p *= 0.015).

To control for false positive results in this codon‐based test, the noncoding portion of the RTGs (after the ancestral stop codon at codon #168) was similarly tested. This 3′ portion of the RTG sequence has been a pseudogene invisible to natural selection throughout the existence of the elephant clade. The branch analysis again showed that a single dn/ds provided an adequate explanation of the data with dn/ds = 0.90 (χ^2^ = 47.61 with 34 *df*, *p *= 0.061), a value not significantly different from 1.0 (χ^2^ = 0.48 with 1 *df*, *p *= 0.489). The test for positive selection at individual codon sites was again highly significant (χ^2^ = 9.59 with 2 *df*, *p *= 0.008), with one codon identified as subject to selection, codon #166 (dn/ds = 2.80; *p *= 0.033; Figure 1). The more permissive test (PAML model M8a and M8) again resulted in a general increase in significance, identifying an additional codon as subject to positive selection, with codon #166 now having an estimated dn/ds = 2.53 (*p *= 0.016), with codon #150 also identified (dn/ds = 2.47, *p *= 0.047).

An alternative to positive selection driving amino acid change is that functional constraint minimizes nonsynonymous substitutions. This possibility predicts a shorter total tree length (i.e., fewer changes) within the first portion of the RTGs (codons 1–88) relative to the 3′ pseudogene region (codons 169‐end). The total tree lengths (per codon) were 1.63 and 1.37, respectively, and for nonsynonymous changes only the estimates were 0.58 and 0.44 per site (all determined using PAML). Both measures were contrary to the prediction of functional constraint.

In summary, comparing the potentially selected 88 codons 5′ of the first frame shift versus the 169 codon “partial pseudogene” 3′ of the ancestral RTG stop codon, both share an overall dn/ds ≈ 1 and, while both yielded a significant signal of positive selection, the ratio of the two chi‐square statistics was essentially identical for the two sequences, *F*(2, 2) = 1.11, *p *= 0.53. This similarity suggests that the evidence for any positive selection in the first 88 codons is likely to be a type I error, perhaps arising from the preponderance of codons evolving neutrally (PAML codeml model M2 classified only 23% of the 88 codons in the dn/ds < 1 group). Furthermore, there was no evidence of selection acting in the opposite fashion to constrain the amino acid sequence of the 5′ potentially functional region of the gene, in comparison to the 3′ pseudogene region.

Sulak et al. (2016) suggested that the expression of the RTGs may be facilitated by the presence of an upstream exon in a retrotransposon (RTE1_LA) within the segmental duplication. RTE1_LA was not associated with all RTGs, and, in agreement with their Figure 3b, it was found that this 237‐bp exon is associated with one cluster of RTGs (#7–19), although within this group it is absent from RTG#9 (and not confirmed in RTG#11 due to a sequencing gap); however, except for the first 55 bp, it is absent from the remaining RTGs (#1–6), and from both the manatee and the hyrax (Table S1; File S2, indicated by the region delimited by “yyy” in the sequence). Hence, this mechanism for RTG expression was absent in the ancestral RTG and during the initial RTG duplications, and was absent during the recent expansion of the RTG group that includes #s2–5 (Figure 2).

Evidence that positive selection may have acted on the first codon of the RTGs could mesh with the possibility that the RTG is transcribed as an internal exon. The first codon of 5 RTGs changed from the ancestral ATG (Figure 1). However, none of these changes were toward the bases typically favored at exon splice sites; furthermore, all RTGs have retained the ancestral GCTGCA immediately preceding the first coding base and this sequence has none of the characteristics typical of the 3′ end of an intron (Mishra et al., 2021).

### 3.4 Why multiple copies of TP53 RTG?

The hypothesis that the elephant RTGs accumulated via natural selection for enhanced cancer suppression is supported by the correlation between the number of RTG copies and body size (Sulak et al., 2016); however, this correlation is to be expected in the absence of any causal relationship. In the absence of selection, neutral changes are expected to fix in a population at a rate equal to the mutation rate. Thus, given a duplication probability of U_dup_ per generation, the rate of fixation of duplicates (in the absence of any effect of natural selection) would be U_dup_ per generation. Assuming that new duplicates have the same duplication probability as the old ones, then the rate of accumulation of new duplicates is expected to accelerate exponentially over time, since, given n duplicates, the fixation probability becomes nU_dup_ per generation. This process predicts a linear relationship between the log of copy number and time, a pattern found by Sulak et al. (2016, their figure 4b). The slope of that relationship, from the origin of the elephant clade, is approximately 4.6 × 10^−8^, which provides a plausible estimate of U_dup_ of 1.1 × 10^−6^, assuming a generation time of 25 years (Wittemyer et al., 2013).

The duplication/drift hypothesis predicts polymorphism for the number of duplications, with the degree of polymorphism depending upon the historical effective population size of elephants. Abegglen et al. (2015) presented elephant sequence data from a new individual that they compared to the pre‐existing genome data (their Figure 2). Their results show substantial differences between the two samples, differences large enough to indicate different duplications; however, without location information it is not possible to separate novel duplications from sequence polymorphism within duplications. Thus, while these sequence data are not definitive, they are consistent with a null hypothesis that the accumulation of the TP53 RTGs has been through a process of duplication followed by random genetic drift.

## 4 DISCUSSION

TP53 is a crucially important tumor suppressor gene that has been named the “guardian of the genome” (Lane, 1992), and the finding of multiple retrogene copies of the gene in the genome of the African and Asian elephants prompted the hypothesis that these copies had been sequentially selected in concert with the increasing body size within the elephant clade (Abegglen et al., 2015; Sulak et al., 2016). In support of the hypothesis, these authors found evidence of enhanced apoptosis in response to double‐stranded DNA damage in both African and Asian elephants, and it was shown that mouse cells transfected with the RTG most abundantly expressed in the elephant (RTG#12) showed a small but significant increase in the induction of apoptosis (Sulak et al., 2016).

In support of these results, Tollis et al. (2021) concluded that there was extensive sequence conservation across the TP53 retrogene loci, conservation that could represent functional constraint; however, the alignment that was provided in the online material indicates that the sequence analyzed was truncated at the 5′ end, spanning from bp#234‐bp#937 (using the elephant TP53) and omitting 209 bp from the remaining 3′ sequence. This raises two concerns: first, it appears that only 62% of the sequence was included in the analysis, and second, the truncation of the 5′ end data means that most of the in‐frame sequence was omitted (see File S1). Here, to test this hypothesis of functional constraint, the two regions of the RTGs (the potentially functional 5′ end and the 3′ pseudogene region) were compared, with the prediction that the 5′ end would show more functional constraint. The data showed that the two regions showed similar constraint (with the numerical results on tree length giving the opposite pattern to that predicted). Tollis et al. (2021) also illustrated functional constraint on p53 with the observation that there were no nonsynonymous changes between the African and Asian elephant TP53; however, there were also no synonymous changes, so the functional constraint argument does not apply (File S1).

Sulak et al. (2016) noted the potential importance of an upstream exon in the expression of the retrogenes. However, this sequence is only present in the scaffolds of RTGs #7–19 (Figure 2; Table S1), a group estimated to have originated about 25 MYA (Sulak et al., 2016). Thus, if this exon is necessary for a beneficial effect of RTGs, then the spread of the initial duplications could not be favored by this effect, but it is consistent with their findings that implicate RTG12 in African elephants (in both adipose and term placental villus tissue) and Asian elephants (in primary dermal fibroblasts). They also found evidence of a very low level of expression of RTG18 in the African elephant and of RTG3 in the Asian elephant, and Abegglen et al. (2015) found that following irradiation of African elephant peripheral blood mononuclear cells and fibroblasts, there was evidence of expression of RTG10/14 (which could not be distinguished) and of RTG5.

Despite these suggestive results there is no direct evidence to support the hypothesis that the size increase in the elephant clade created the selection pressure that drove a progressive increase in RTG copies of TP53. Instead, the evidence supports the null hypothesis that the duplicates accumulated through recurring duplication and drift. In summary, this evidence is: (i) the TP53 transcript was truncated before the origin of the elephant clade, and even more so before the first RTG duplication within the clade, excluding the possibility that there was ever selection on duplicate RTGs that produced fully active p53; (ii) that there is no evidence that the potentially transcribed 5′ portion of the RTGs has been subject to either directional or stabilizing selection since it has a dn/ds ≈ 1, typical of a pseudogene, a view reinforced by the finding that it has evolved similarly to the 3′ portion that has been acting as a pseudogene for more than 40 million years (based on the time estimates of Sulak et al., 2016); and (iii) the exponential (i.e., log linear) accumulation of duplicates over time is consistent with a neutral model of duplication and random fixation. Supporting this last possibility is the observation that the correlation of duplicate number with body size breaks down in the case of the forest elephant (*Loxodonta cyclotis*). Tollis et al. (2021) estimated that there are more than 20 RTGs in the forest elephant's genome. This is consistent with the drift hypothesis, since, even though it decreased in body size after splitting from the lineage of the African elephant, it has continued to accumulate RTGs.

It is clear that much of the functional potential of the RTGs has been lost. 74% (14/19) of the RTGs have ≤88 amino acids (relative to the canonical p53) corresponding to the first 98 amino acids of p53, less 10 that have been deleted. As a result, any potential transcript is terminated before the p53 region involved in DNA binding, and the remaining 5, although they have part of this region, they all lack the dimerization site and the specific DNA‐binding site (Table S1). RTG#7 has retained the longest region of 5′ homology with the p53 protein (157 codons), but it lacks a start codon. The manatee and hyrax RTGs also show an extreme loss of potentially functional protein (Table S1).

Estimates of the degree of adaptive change needed to keep the incidence of cancer in elephants at a low level (Table 1) are consistent with the selection in the elephant clade favoring a gain of the equivalent of 1–2 tumor suppressor genes. This predicted gain could have involved an increase in p53 activity; however, for added tumor suppressor gene activity to be effective against the increased lifetime burden of somatic mutation experienced by elephants, each tumor suppressor gene needs to act independently, so that disabling driver mutations eliminating one tumor suppressor gene do not disable others. For example, two functional copies of TP53 could work in concert, since it would require four driver mutations to eliminate p53 activity. The extreme truncation of the retrogene copies plus the absence of evidence for selection to conserve the truncated region argues against their independent tumor suppressor activity. However, if the truncated RTGs maintain p53 activity indirectly by enhancing the effectiveness of the single TP53 gene, which seems to be the only plausible mode of action (Abegglen et al., 2015; Sulak et al., 2016), then mutations disabling TP53 would simultaneously remove any benefit due to the activity of the RTGs.

So how could selection favor such an indirect effect of the truncated RTGs? One possibility is that the observed enhanced sensitivity of elephant cells to DNA damage (Abegglen et al., 2015; Sulak et al., 2016) reduces the “effective” somatic mutation rate by removing cells with an elevated risk of carrying driver mutations. Reduction in somatic mutation rate is potentially an important mechanism of increasing cancer suppression, and very large reductions are favored (Table 1). It is certainly possible that at some point prior to the split of African and Asian elephants about 7 MYA (Rohland et al., 2010), a positive effect on p53 activity became a characteristic of at least one duplicate (e.g., RTG#12), and that perhaps this effect has been retained by selection. This role may have also favored the recruitment of a duplicated LIF gene (Vazquez et al., 2018). However, the dependence of any RTG benefit on the functioning of the canonical TP53 suggests it is probable that other as yet undiscovered mechanisms of tumor suppression have also evolved in the elephant lineage, perhaps recruited from the array of cancer‐related genes that have been duplicated in the elephant genome (Vazquez & Lynch, 2021). Similarly, Ferris et al. (2018) identified a region of accelerated evolution in the elephant genome that could be related to changes in the response to DNA damage.

The investigation of cancer suppression in nonhuman animals can be expected to yield valuable novel insight into cancer prevention (Nunney et al., 2015; Ujvari et al., 2017). It can be seen that there is not a large difference between the degree of cancer suppression expected to evolve in elephants and humans (Table 1), but it is important to bear in mind that these two lineages have adapted independently from smaller and shorter lived ancestors. Hence, the adaptations present in elephants may be quite different from those protecting humans from cancer. Thus, any enhanced effect of p53 and other mechanisms of cancer suppression in elephants and in other large and/or long‐lived animals may be very valuable in gaining insight into the array of mechanism that natural selection has identified to reduce the risk of cancer. Searching for such mechanisms is difficult, but valuable approaches have been developed such as looking for genomic regions of accelerated evolution (Ferris et al., 2018). Understanding the array of mechanisms that evolution has exploited to reduce the risk of cancer during the reproductive life of big and/or long‐lived animals has a great potential benefit for humans. We already know from the discovery of antibiotics just how valuable the defense mechanisms evolved in nature can be.

## CONFLICT OF INTEREST

None.

## Supporting information

Supplementary Material

## Data Availability

All sequence data are available publicly at NCBI, with the alignments used provided in Files S1 and S2.
