# Supplementary material for: Cancer suppression and the evolution of multiple retrogene copies of TP53 in elephants: A re‐evaluation
Source: Evol Appl. 2022 Apr 25;15(5):891–901. doi: 10.1111/eva.13383 (PMC9108310; doi:10.1111/eva.13383)
Supplement: Supplementary file 1 — Supplementary Material [file EVA-15-891-s001.zip › eva13383-sup-0003-TableS1.pdf]

Supplementary Table 1: Features of the RTGs of the African elephant showing (i) the extent of the p53 conservation, and (ii) conservation of the 50kb upstream region of the RTGs relative to the two ancestral sequences of hyrax and manatee.

Grey bolded cells show the maximum number of codons homologous with the 390 codons of the canonical p53 that could be potentially transcribed.

Regions of the p53 protein are defined from Sulak et al (2016).

Positions of relatively continuous upstream alignment with the hyrax sequence are measured on the hyrax RTG scaffold (from after the start codon: "xxx" in Suppl. File #2). Two non-contiguous (i.e. isolated regions) of upsteam alignment are also noted in the last two columns.

	start codon?	codon position of corrected frameshifts (FS) & deletes	frameshift after codon number	Number of codons before a stop	percent of homologous TP53 codons	potential upstream exon of 237bp - see text	end of upstream align (on hyrax RTG scaffold)	length of isolated 5' align with manatee RTG at 31882 (manatee scaffold)	length of isolated 5' align with hyrax RTG at 34594 (hyrax scaffold)
Af. elephant TP53	yes	n/a	n/a	**390**	100.0	n/a	n/a	n/a	n/a
pre-elephant RTG	yes	12aa FS (codon212-223)	**242**	334	62.1	-	-	-	-
**Potential trancript terminated within the DNA binding domain, with no dimerization site, no DNA binding site**									
pre-duplication of elephant RTG	yes	5aa del (after codon77)	**157**	167	40.3	-	-	-	-
RTG#1	no	n/a	n/a	**5**	1.3	no	29572[a]	none	455
RTG#2	yes	5aa del (after codon77)	n/a	**134**	34.4	no	18698	3763	429
RTG#4	yes	5aa del (after codon77)	n/a	**134**	34.4	no	18698	>2296	none
RTG#5	yes	5aa del (after codon77)	n/a	**134**	34.4	no	18698	3809	none
RTG#6	yes	5aa del (after codon77)	n/a	**123**	31.5	no	8725	none	none
RTG#7	no	5aa del (after codon77)	**157**	167	40.3	yes	8725	none	none
**Potential trancript terminated before the DNA binding domain starts**									
RTG#3	yes	5aa del (after codon77)	n/a	**79**	20.3	no	>6298	no data	no data
RTG#8	yes	10aa del (after codon72)	**88**	210	22.6	yes	8725	none	none
RTG#9	no	10aa del (after codon72)	**88**	210	22.6	no	>2123	no data	no data
RTG#10	yes	10aa del (after codon72)	**88**	162	22.6	yes	>6282[b]	none	none
RTG#11	yes	10aa del (after codon72)	**88**	203	22.6	no data	8725	none	none
RTG#12	yes	10aa del (after codon72)	**88**	180	22.6	yes	>4141	no data	no data
RTG#13	no	10aa del (after codon72)	**88**	180	22.6	yes	8725	no data	no data
RTG#14	yes	10aa del (after codon72)	**88**	210	22.6	yes	>5971	no data	no data
RTG#15	yes	10aa del (after codon72)	**88**	210	22.6	yes	8725	no data	no data
RTG#16	no	10aa del (after codon72)	**88**	203	22.6	yes	8725	no data	no data
RTG#17	yes	10aa del (after codon72)	**88**	111	22.6	yes	8725	no data	no data
RTG#18	yes	10aa del (after codon72)	**88**	111	22.6	yes	8725	none	none
RTG#19	yes	10aa del (after codon72)	**88**	210	22.6	yes	8725	no data	no data
Manatee RTG	no	25bp del/FS (after codon3)	**3**	5	0.8	no	-	-	-
Hyrax RTG	yes	4aa del (after codon44)	**45**	128	11.5	no	-	-	-

a: Alignment gap of 7240bp (hyrax scaffold) starting at 9856; b: alignment ends at a region of unassigned bases ("N"s), but scaffold continues
